# The Pair Test: A computerised measure of learning and memory

**DOI:** 10.3758/s13428-020-01470-9

**Published:** 2020-09-09

**Authors:** Sarah Buck, Filipa Bastos, Torsten Baldeweg, Faraneh Vargha-Khadem

**Affiliations:** 1 Cognitive Neuroscience and Neuropsychiatry Section, Developmental Neurosciences Research and Teaching Department, UCL Great Ormond Street Institute of Child Health, London, UK; 2 Great Ormond Street Hospital for Children, National Health Service Trust, London, UK; 3 Unit of Paediatric Neurology and Neurorehabilitation, Woman-Mother-Child Department, Lausanne University Hospital, Lausanne, Switzerland

**Keywords:** Memory, Learning, Paired-associate learning, Test, Assessment, Children

## Abstract

There is increasing interest in the assessment of learning and memory in typically developing children as well as in children with neurodevelopmental disorders. However, neuropsychological assessments have been hampered by the dearth of standardised tests that enable direct comparison between distinct memory processes or between types of stimulus materials. We developed a tablet-based paired-associate learning paradigm, the Pair Test, based on neurocognitive models of learning and memory. The aims are to (i) establish the utility of this novel memory tool for use with children across a wide age range, and (ii) examine test validity, reliability and reproducibility of the construct. The convergent validity of the test was found to be adequate, and higher test reliability was shown for the Pair Test compared to standardised measures. Moderate test–retest reproducibility was shown, despite a long time interval between sessions (14 months). Moreover, the Pair Test is able to capture developmental changes in memory, and can therefore chart the developmental trajectory of memory and learning functions across childhood and adolescence. Finally, we used this novel instrument to acquire normative data from 130 typically developing children, aged 8–18 years. Age-stratified normative data are provided for learning, delayed recall and delayed recognition, for measures of verbal and non-verbal memory. The Pair Test thus provides measures of learning and memory accounting for encoding, consolidation and retrieval processes. As such, the standardised test results can be used to determine the status of learning and memory in healthy children, and also to identify deficits in paediatric patients at risk of damage to the neural network underlying mnemonic functions.

**Electronic supplementary material:**

The online version of this article (10.3758/s13428-020-01470-9) contains supplementary material, which is available to authorized users.

## Introduction

### Memory processes: Encoding, learning, recall and recognition

Episodic memory comprises the ability to encode, consolidate and retrieve past events along with their contextual details. During encoding, information is perceived and transformed into a mental representation. It is difficult to quantitatively measure encoding, and as a result, this introspective process is not well understood. Practically, however, encoding can be measured through immediate retrieval of a stimulus after a single presentation, thus providing a measure of encoding that is available to retrieval processes. However, it remains difficult to distinguish between the processes of encoding and retrieval, which are both required at this stage.

There are two cognitive processes that accompany retrieval of information: recollection and familiarity (Mandler, 1980). Recollection refers to the reliving of past episodes with vivid and detailed retrieval of the event, whereas familiarity is associated with a sense that an item was previously encountered, but without its contextual details (Tulving, 1985). From a quantitative perspective, it is difficult to tap into the introspective processes of recollection and familiarity (Gardiner, 2001), but neurocognitive tests can provide measures of recall and recognition to assess these processes, respectively. Thus, recall and recognition processes are used as proxy for the conscious processes of recollection and familiarity. *Recall* refers to the ability to bring back to mind encoded and/or consolidated representations, whereas *recognition* reflects the ability to identify presented items as previously encountered and therefore familiar. A recognition test provides a measure of recognition (i.e. awareness that the stimulus has been encountered before), although recollection might also occur alongside a sense of familiarity. It is important to be aware of this, as a score obtained with a recognition-based test might reflect the dual processes of recollection and familiarity.

In the experimental and clinical settings, the ability to learn arbitrary associations is often assessed (Roediger and Nestojko, 2015). Such associative learning reflects the ability to form arbitrary associations between items and bind their features into a new integrated percept. After learning has taken place, maintenance of the bound representation occurs as a function of elapsed time and consolidation, and retrieving that same information from memory can occur through recall and/or recognition.

#### Neural substrates

The processes of encoding, learning, recollection and familiarity are supported by distinct, yet interactive, neural substrates within the temporal lobes. The hippocampus plays a critical role in encoding trial-unique events (Squire, 1992; Steele and Morris, 1999; Bast et al., 2005). The hippocampus is involved in forming and retrieving novel associations (Konkel and Cohen, 2009), as required during paired-associate learning tasks (Eichenbaum et al., 1994; Brown and Aggleton, 2001; Davachi, 2006; Hannula et al., 2006; Manns and Eichenbaum, 2006; Diana et al., 2007). Similarly, it has been proposed that retrieval processes of recollection and familiarity are anatomically distinct (see Yonelinas, 2002, for a review). Whereas the hippocampus is involved in recollection processes, it is thought that familiarity processes rely on other medial and inferior temporal regions, such as the perirhinal, entorhinal and parahippocampal cortices (Davachi et al., 2003; Diana et al., 2007; Eichenbaum et al., 2007). As a result of the distinctive processes outlined above, and of the selective underlying neural substrates supporting these processes, neuropsychological tests should aim to provide specific measures of encoding, learning, recall and recognition abilities, and by implication, indications of the functionality of the neural substrates underlying these specific components of memory.

#### Developmental trajectories

There are distinct developmental trajectories for the processes outlined above. Evidence for single-item recognition memory is present in the first few days of life (Fagan, 1970), and several studies have demonstrated that familiarity judgement is age-invariant from the age of eight onwards (Naus et al., 1977, although see Newcombe et al., 1977; Davidson and Hoe, 1993, for an alternative view). On the other hand, the ability to form and learn relational associations between stimuli develops somewhat later in life, first emerging around the age of 5 or 6 years (Peterson, 2002). Moreover, recollection processes show more developmental changes throughout childhood and adolescence (Ghetti and Angelini, 2008; Bjorklund et al., 2009; Jabès and Nelson, 2015; Bauer et al., 2017; Alibran et al., 2018; Rollins and Riggins, 2018), with age-related improvements in recollection of contextual details. The intrinsic role of the hippocampus in learning and retrieval and the extended trajectory of hippocampal development (Gogtay et al., 2006) reflects the wide range of individual variation in both typical and clinical populations. As a result of these developmental changes, it is critical for neuropsychological tests to detect variations in stages of memory development as a function of increasing age.

### Lateralisation of memory functions

Behavioural evidence for lateralisation of memory relies on hemisphere-dependent deficits in relation to type of stimulus material. In adults with unilateral temporal lobe pathology, such as temporal lobe epilepsy (TLE), material-specific memory impairments are often reported, with verbal memory deficits in patients with left TLE and visual memory deficits in patients with right TLE (Jones-Gotman et al., 2000; Helmstaedter et al., 2003; Jones-Gotman et al., 2010). The pattern of complementary impairments caused by unilateral lesions reflects hemispheric specialisation of function and provides strong clues about the organisation of memory in the healthy mature brain. In contrast to adults, material-specific deficits are not as clearly side-dependent in children with early-onset brain pathology (Helmstaedter and Elger, 1998; Gleissner et al., 2005; Willment and Golby, 2013; Hamberger et al., 2019).

In children, lateralisation of function, especially for language and verbal memory functions, does not emerge before the age of 5 years (Vargha-Khadem et al., 2000), and gradually becomes established during development (Vargha-Khadem and Polkey, 1992; Vargha-Khadem et al., 1994). Thus, during the early stages of infancy and childhood, speech/language and verbal learning and memory functions in general may be bilaterally represented, but become progressively lateralised to the left, with reduced contribution from the right hemisphere.

Importantly, early brain pathology interferes with the normal processes of circuit specialisation and hemispheric lateralisation (Willment and Golby, 2013), which are sacrificed to facilitate neural plasticity and, in turn, rescue cognitive functions (Cacucci and Vargha-Khadem, 2019). Compensatory reorganisation of function is facilitated by greater potential for plasticity following an injury in younger patients, thereby impeding, or abolishing the normal lateralisation process (Cacucci and Vargha-Khadem, 2019). Early-onset pathology and efficient neural plasticity therefore result in a pattern of non-specialised hemispheric organisation and, consequently, a diffuse representation of cognitive functions (Vargha-Khadem et al., 2000).

### Pitfalls of current standardised tests

Studies examining material-specific impairments associated with unilateral brain pathology show inconsistent findings, possibly due to limited hemispheric specialisation for memory in childhood pathology. The tests designed to measure lateralised effects in the developing brain may also have shortcomings. Confounds in the measurement of memory processes that these tests purport to assess, as well as the modality of items to be remembered, may hamper clear comparisons between verbal and visual tests (Hamberger et al., 2018).

First, standardised verbal and visual memory tasks often assess distinct cognitive processes, wherein verbal memory is usually tested through recall, and non-verbal (i.e. visual) memory through recognition. For the purpose of this manuscript, we refer to “non-verbal” stimuli to describe visual material presented in the visual modality. Second, differences in the modality of presentation of verbal and non-verbal material could also contribute to inconsistent findings. Verbal tasks are usually presented in the auditory modality (e.g. spoken words), whereas non-verbal tasks are presented in the visual modality (e.g. designs). This is a confound that overrides the quality of the stimulus material. Information in the auditory modality is received in temporal order, whereas the visual modality is more prone to configural processing, at least for static stimuli. In this respect, it is important that stimuli across both tasks are presented in the same sensory modality, for instance, assessing verbal memory in the visual modality (i.e. written words) for better comparisons with non-verbal visually presented memory tasks. In addition, differences in task difficulty between input modalities may hamper clear investigation of lateralisation of memory deficits. Third, studies often compare verbal associative memory (i.e. word pairs) with single-item visual memory (e.g. one complex figure). Distinct neural mechanisms subserve these processes, whereby the hippocampus contributes to associative memory, and other non-hippocampal medial temporal regions contribute to single-item memory (Eichenbaum et al., 1994; Henke et al., 1999; Brown and Aggleton, 2001). Finally, standardised tests of visual memory may be insensitive to right hemisphere pathology, which may allow some level of verbal labelling of pictures. The lack of specificity of non-verbal stimuli may therefore hinder the test’s ability to capture impairments in visual memory which cannot reliably distinguish between pathology in the left versus right hemisphere (Lee et al., 2002; McConley et al., 2008). Overall, the nature of unbalanced standardised tests has made it difficult to investigate the presence or absence of lateralisation of function associated with unilateral pathology in the developing brain. More balanced and controlled paradigms are required to investigate this further.

### The Pair Test

The Pair Test is a computerised paired-associate learning paradigm that was developed for the assessment of hippocampal-dependent learning and memory processes, consistent with theoretical knowledge of neural substrates supporting encoding, learning, recall and recognition. It is composed of five subtests, each consisting of paired-associate learning (verbal and non-verbal) over three consecutive trials, as well as recall and recognition of those pairs after a short delay.

This tool was designed to be appropriate for a wide age range. Moreover, the Pair Test allows for comparison of distinct learning and memory processes within the same material type (verbal or non-verbal), and also optimises comparison of memory for verbal versus non-verbal information. Finally, this tool controls for the input modality (auditory versus visual) and for the levels of semantic structures of information, as discussed below.

The input modality of information to be remembered is controlled in the Pair Test. Long-term auditory memory requires subvocal reproduction of speech sounds by means of the oromotor system and, in this respect, is closely related to speech (Schulze et al., 2012). Therefore, it is possible that children who present with language impairments may also exhibit auditory long-term memory deficits. Tests of auditory verbal memory are thus not comparable with those of non-verbal memory which are presented in the visual modality, and may not be suitable for the examination of lateralisation of memory function. The Pair Test not only enables examination of memory lateralisation whilst controlling for the input modality, but also permits direct comparison of modality of presentation (auditory versus visual) for verbal information.

The levels of semantic structure of information to be remembered are also controlled in the Pair Test. Whereas memory for familiar stimuli (words and objects) can rely on previously stored representations, memory for non-semantic stimuli (pseudowords and abstract shapes) must rely on newly established representations. Non-semantic subtests therefore push the boundaries of new learning, and the establishment of these new representations may depend on mnemonic strategies at encoding. In that respect, non-semantic tests may serve as more sensitive indicators of damage to critical regions subserving memory.

The paradigm is based on four balanced subtests presented in the visual modality (intra-modal), examining the effects of material type and level of semantic structure. Importantly, there is also one cross-modal subtest (verbal information presented in the auditory modality) to evaluate the differential effects of modality on verbal learning and memory.

The aim was to establish the utility of this novel memory tool for use with children across a wide age range. We were also interested in examining the contributions of intellectual ability and/or executive functions to performance on the Pair Test, compared to other standardised tests. Finally, this study will also examine test validity, reliability and reproducibility of the construct.

## Methods

### Participants

One hundred and thirty typically developing children and adolescents between the ages of 8 and 18 years were recruited for the study (M = 13 years, SD = 3). These children were approached through East London schools, and were all English-speaking with no history of psychiatric or neurological disorder. Participants were not excluded based on learning disabilities (identified through parental questionnaires), such as dyslexia or ADHD, in order to provide a better representation of the general population. In the full cohort, five children had learning difficulties (attention difficulties, *n* = 2 and dyslexia, *n* = 3), but nonetheless had normal IQ. Informed written consent was obtained prior to study start from parents for participants under 18 years old, and from participants themselves if they were 18 years old. The cohort was composed of 30 male and 100 female participants.

### Socioeconomic status

Socioeconomic status (SES) was determined based on the participants’ postcode using the Index of Multiple Deprivation from the UK Ministry of Housing, Communities and Local Government. Deprivation deciles range from most deprived (score of 1) to least deprived (score of 10). Participants in the present cohort had SES scores across the whole range (M = 4, SD = 2, min = 1, max = 10).

### Neuropsychological assessment

General intellectual functioning was assessed using the Wechsler Abbreviated Scale of Intelligence Second Edition (WASI-II) (Wechsler, 2011). This test provides measures of intellectual ability for full-scale IQ (M = 104, SD = 10), verbal IQ (M = 106, SD = 10) and performance IQ (M = 100, SD = 11). No participants had standard scores below 70.

Memory ability was assessed using the Children’s Memory Scale (CMS) (Cohen, 1997). For the purpose of this study, only two subtests of the CMS were administered: the Dot Locations and Word Pairs. These subtests provide measures of word pair learning (M = 92, SD = 17), word pair delayed recall (M = 96, SD = 15), dot location learning (M = 103, SD = 15) and dot location delayed recall (M = 104, SD = 14).

### The Pair Test

#### Five subtests

The Pair Test is composed of five subtests, controlling for material (verbal versus non-verbal), input modality (auditory versus visual) and conceptual components of items (semantic versus non-semantic, where semantic items are familiar and non-semantic items rely on newly established representations; see section 1.4 for a definition of those terms) (see Table 1).

**Table 1:**
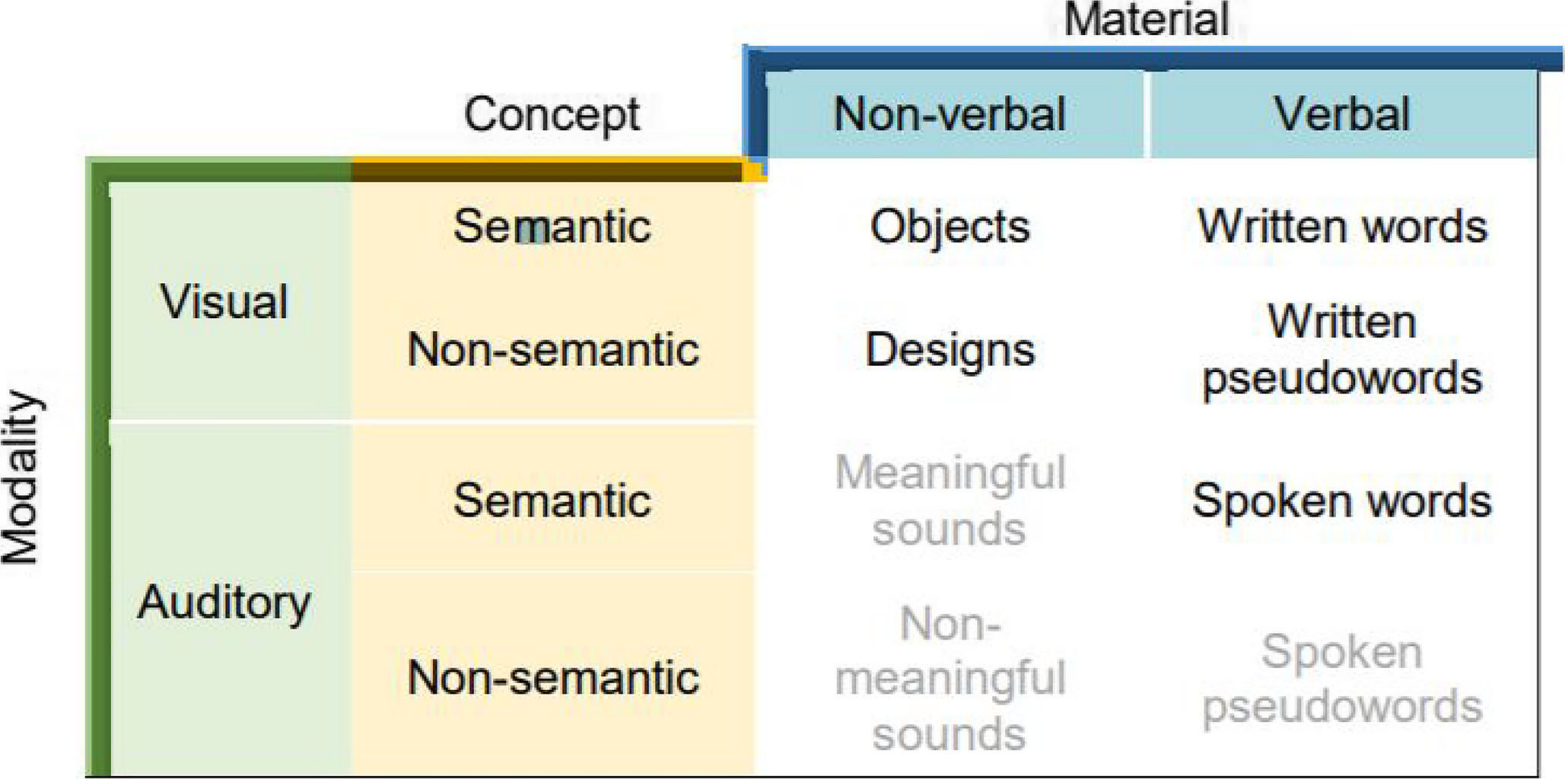
Overview of the experimental paradigm

The five subtests consist of paired-associate learning of Spoken Words, Written Words, Objects, Abstract Designs, and Pseudowords. The remainder of the stimulus categories from Table 1 were not developed because of the difficulty retrieving sounds through the process of recall. However, the five stimulus categories selected for the development of the Pair Test allow comparison between the three variables of interest.

#### Stimulus material

The stimuli with access to semantic labels (i.e. objects and written words) were selected from the MRC Psycholinguistic Database (Wilson, 1988), and were matched to each other on age of acquisition (Kuperman et al., 2012), verbal frequency (Brown, 1984), word length, concreteness, familiarity and imageability. For the Object task, 60 object stimuli were selected from Snodgrass’ original data set based on concept familiarity and visual complexity (Snodgrass and Vanderwart, 1980). For these stimuli, concept familiarity ranged from 1.4 to 4.95 and visual complexity ranged from 1.10 to 3.90 on a five-point rating scale (where 1 indicates simple and 5 indicates very complex). The pseudoword items were composed of monosyllabic and bi-syllabic pronounceable non-words and were matched to the words in terms of the number of syllables. The design stimuli were composed of black and white abstract, but reproducible, line drawings. Each subtest was composed of 10 pairs, amongst which 8 were composed of unrelated items (hard pairs) and 2 were composed of related items (easy pairs).

#### Two parallel versions

For each of these subtests, two versions were created using different stimuli to enable administration of parallel versions to the same participants at two different time points (e.g. before and after intervention). The stimuli selected for Spoken Word, Written Words and Object subtests were equivalent across the two versions.

#### Tablet-based application

An application was developed using the MIT App Inventor 2 software for the presentation of the stimuli and the recording of the responses. Administering the memory subtests with a tablet makes the testing procedure more engaging and child-friendly, and also allows a more controlled administration process.

#### Learning and memory processes

##### Learning

**Fig. 1:**
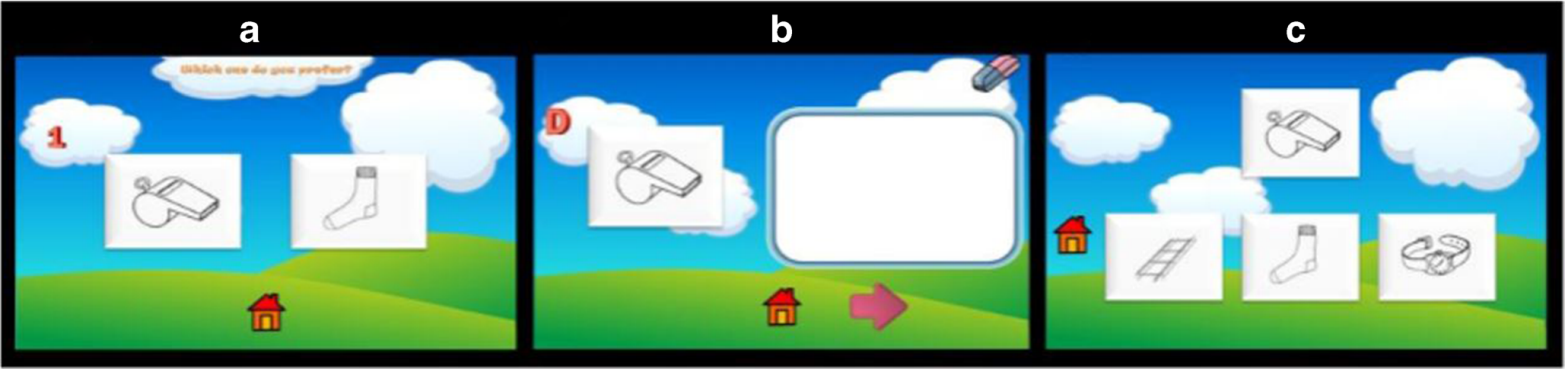
***a*** Encodin*g.* ***b*** Cued recall. ***c*** Recognition

The list of 10 stimulus pairs was presented to the participants, who were asked to make a preferential judgement by clicking on the preferred item (Fig. 1). This was to ensure the encoding of each pair within each subtest, and the use of the same procedure across the five subtests. After the last pair of the list was presented, participants were immediately presented with the stimulus appearing on the left-hand side of the screen for each pair and asked to recall by drawing (for non-verbal items) or writing (for verbal items) the stimulus that was paired with it (Fig. 1). This encoding-cued recall cycle was carried out three times in succession for each subtest to establish a learning curve for each participant on that subtest. No feedback was given on their performance.

##### Delayed recall

A final cued recall trial was administered after a 15-minute delay, where participants were presented with a cue, i.e. the first stimulus item of the pair, and were asked to remember the item that was paired with it. This was performed for each pair of each subtest. During this delay period, the learning phase of another task took place.

##### Delayed recognition

In the forced-choice recognition stage, participants were presented with the first stimulus of each pair (i.e. the cue) and asked to pick from three choices the target associate that was paired with the cue (Fig. 1). Amongst the three choices, there were two distractors: one new stimulus and one familiar stimulus that was among the list to be remembered, as part of a different pair. With this paradigm, the distractors cannot be rejected purely on the basis of familiarity, and recall of the association is required to make recognition judgements.

#### Procedure of administration

The administration of the five subtests was counterbalanced between and within material type (verbal and non-verbal mixed in order to prevent interference). Moreover, the order of administration of tasks was randomised between participants (respecting the counterbalance of material type) using a random number generator so that participants did not perform the tasks in the same order. The administration of the whole paradigm takes about 1 ½ hours, and took place at the UCL Great Ormond Street Institute of Child Health or at the participants’ school.

### Statistical analyses

Analyses were performed on raw data, rather than on age-controlled standard scores, in order to be able to capture age-related differences. These analyses were conducted using SPSS version 25 software.

#### Test validity

Participants were administered either version A or B; therefore, a between-group design was used to examine the comparability of the two versions. A mixed-ANOVA was performed, with test versions as between-subject factor, and cognitive measures (learning, delayed recall and delayed recognition) and five subtests as within-subject variables. This allows us to examine (i) the feasibility of combining the two versions for subsequent analyses, and (ii) the utility of this tool for comparable assessment across two time points. Another mixed-ANOVA was performed with sex as between-subject factor.

Convergent validity was also examined. This type of validity is the degree to which two measures that are assumed to measure the same construct are related. We can explore convergent validity between the CMS and the Pair Test and assess to what extent they measure the same construct of learning and memory. Pearson correlation analyses between each subtest of the Pair Test and the two subtests of the CMS were computed for measures of learning, delayed recall and recognition separately, with the full-scale IQ (FSIQ) partialled out to account for levels of general cognitive ability.

#### Test reliability

Reliability analysis was computed on the Pair Test to determine how closely related the subtests are and how strongly each subtest is associated with the memory component that the paradigm measures. An exploratory factor analysis was performed to understand the structure of the subtests from the two paradigms (i.e. the Pair Test and the CMS). Exploratory factor analysis identifies the least number of factors which can account for the common variance of variables. This analysis was conducted for measures of learning, delayed recall and recognition, separately. Exploratory factor analysis consists of separating the variables into factors based on statistical measures. A factor loading is produced for each subtest of the two paradigms as an indication of how strongly each subtest is associated with the factor. Factor rotation was applied to best discriminate between factors. More specifically, “direct oblimin rotation” was applied due to the expected correlations between factors. Eigenvalues (i.e. indication of the importance of the factor) greater than 1 were used as a threshold to determine the number of factors to retain. Although this is an inclusive method which may produce spurious factors, the threshold was defined based on the scree plot, which reduces the risk. Moreover, Cronbach’s alpha was calculated on the Pair Test paradigm to assess the internal consistency of the construct.

#### Test–retest reproducibility

A subgroup of 19 participants was invited back for a second assessment about one year after the first, and was administered the Pair Test again (the alternative test version used in the first assessment). The reproducibility of the paradigm (or alternate form reliability) was determined based on the consistency of performance across the two time points (within-subject design), with a mean interval of 14 months (95% CI: 9.6–18.2), using different test versions. Interclass correlation coefficient (ICC) using a two-way mixed-effects model with absolute agreement was applied on the mean measurements of chosen test measures. This is a robust reproducibility method, widely used in neuropsychology literature (Koo and Li, 2016; Parsons et al., 2019).

#### Capturing a wide range of abilities

The ability of the Pair Test to capture a wide range of abilities was verified by calculating the percentage of participants with floor and ceiling performance in each subtest of the Pair Test. A floor performance refers to a 0% correct score achieved at the third trial, in which case the task is too difficult. A ceiling performance refers to a 100% correct score at the first trial, in which case the task is too easy.

#### Developmental changes

The ability of the Pair Test to capture developmental changes in learning and memory was examined with partial Pearson correlations between the variables of age and measures of learning, delayed recall and delayed recognition, with FSIQ partialled out.

#### Variance explained by FSIQ

Although it is expected that intellectual status contributes to learning and memory functioning in typically developing children, regression analyses were conducted to examine the extent of this contribution to performance on the subtests of the CMS and the Pair Test. Simple linear regressions were calculated to predict memory scores based on FSIQ. These regressions were computed separately for the Pair Test and the CMS, on measures of learning, delayed recall and delayed recognition, separately.

First, we checked for (i) homoscedasticity of variance and (ii) whether the residuals of the outcome predicted were normally distributed. Supplementary Figure 1 shows a scatterplot of the values of the residuals against the values of the outcome predicted by the model. The plots show no systematic relationship between the errors in the model and what the model predicts, and thus the assumption of homoscedasticity is met, for both the Pair Test and the CMS. In addition, the histograms show normally distributed residuals (Supplementary Figure 1). *R*^2^ is the squared correlation between values of memory scores predicted by the model and the values observed in the data. This value provides a measure of how well the memory scores can be predicted by the FSIQ.

### Standardisation of raw scores

For each subtest of the Pair Test, standardisation of raw scores was performed for five separate age groups (group 1: 8–9, group 2: 10–11, group 3: 12–13, group 4: 14–15, group 5: 16–18 years old). Standardisation was conducted on five age groups from 8 to 18 years to account for changes in cognitive developmental profiles as a function of age. The division into five age bands allows a balanced composition of groups with equivalent numbers of individuals in each age group.

Raw scores were converted to *z*-scores by subtracting the mean and dividing by the standard deviation for each age group separately. Those scores were then multiplied by 15, and 100 was added to compute scores analogous to Wechsler scores with a mean of 100 and a standard deviation of 15. A score of 100 therefore reflects the average performance of a given age group. A case-wise deletion of missing data (*n* = 1%) was employed in the computation of the standard scores.

### Index scores

Index scores were derived from the average of each participant’s raw scores on the relevant subtests (Table 2), which were then converted to standard scores as per the procedure described in 2.6. The derivation of index scores allows for the investigation of different variables of interest, i.e. material type, access to semantic label and modality of presentation. In addition, despite not being index scores per se, performance on the Spoken Words and Written Words subtests can be compared to provide an indication of modality differences (auditory and visual modality, respectively).

**Table 2:** Index scores and the subtests they comprise

Index scores	Subtests that the index comprises
Verbal material	Written Words and Pseudowords
Non-verbal material	Objects and Designs
Semantic	Written Words and Objects
Non-semantic	Pseudowords and Designs

Two additional general indices were computed: a “general learning/recall” index derived from the average learning and delayed recall scores across the five subtests, and a “general recognition” index derived from the average recognition scores across the five subtests. These indices provide general memory measures, irrespective of material type, modality or level of semantic structure.

### Differences between index scores

An important consideration for interpreting the performances across domains is the amount of difference between different standard scores. The minimum difference between any pair of scores required for statistical significance was computed. Because very small variance is observed between the different age groups, the values are shown for all age groups combined. To obtain those values, the first step was to calculate the standard deviation of the scores’ paired difference (SDy), for a measure of variability. Correlation between the two scores (ry.x) was then calculated, for a measure of reliability. The standard error of measurement of the difference was then calculated using the formula below (Harvil, 1991), where *N* = 130.

$$ {SE}_{Mdiff}={SD}_{x.y}\sqrt{\left(1-{r}^2\right)\frac{N-1}{N-2}} $$

Those values were then multiplied by a factor of 1.96 to yield the amount of difference that is statistically significant at *p* ≤ 0.05. In addition, the frequency of the difference in the standardisation sample is represented in separate tables to provide an indication of how frequently such discrepancy is observed in the general population (see manual). Discrepancy between scores is found in the document entitled “Significance of discrepancy between scores” (see section 7).

## Results

### Test validity

A mixed-ANOVA was conducted, with test version (2: A and B) as between-subject factor and subtest (5: Spoken Words, Written Words, Pseudowords, Objects and Designs) and measure (3: learning, delayed recall and delayed recognition) as within-subject variables. There was no significant effect of test version (*p* = 0.307), nor was there a significant interaction between test version and subtest (*F*(1,115) = 0.111, *p* = 0.739) or between test version, subtest and measure (*F*(1,115) = 1.19, *p* = 0.279). An independent-samples *t*-test showed no significant age difference (*t*(129) = -0.336, *p* = 0.737), and a chi-square test showed no significant gender difference between the two groups (*x*^2^(1,*N* = 130) = 0.582, *p* = 0.446). Memory scores from the two versions were therefore collapsed for subsequent analyses.

A mixed-ANOVA was also conducted with sex as between-subject factor. This showed no significant effect of sex (*F*(1,115) = 1.21, *p* = 0.273) or a significant interaction between sex, subtest and measure (*F*(1,115) = 3.13, *p* = 0.079).

Convergent validity was also examined, and results are shown in Table 3 for measures of learning, delayed recall and delayed recognition. We hypothesised that (i) scores on the Dot Locations subtest would be more closely related to visual subtests of the Pair Test than verbal subtests, due to the type of stimulus material to remember, and that (ii) all subtests of the Pair Test would generally be more closely related to the Word Pairs subtest than the Dot Locations subtest of the CMS.

**Table 3:** Correlation coefficient between Pair Test and CMS, for learning, delayed recall and delayed recognition scores

	Learning			Delayed recall			Delayed recognition	
	Dot Locations	Word Pairs	Fischer’s test *z*	Dot Locations	Word Pairs	Fischer’s test *z*	Dot Locations	Word Pairs
Spoken Words	0.24*	0.39**	−1.5*	0.12	0.36**	−2.2**	NA	0.17
Written Words	0.23*	0.47**	−2.5**	0.14	0.38**	−2.2**	NA	0.34**
Objects	0.31**	0.38**	−0.7	0.21*	0.38**	−1.6*	NA	0.32**
Designs	0.29**	0.46**	−1.8*	0.18	0.48**	−2.9**	NA	0.24**
Pseudowords	0.35**	0.32**	0.31	0.15	0.44**	−2.8**	NA	0.40**

**Correlation is significant at the 0.01 level (two-tailed)

*Correlation is significant at the 0.05 level (two-tailed)

Moderate correlations are observed between the subtests scores involving cued recall from the Pair Test and Word Pairs from the CMS (*p* < 0.001). Low and non-significant correlations are found between the subtest scores of the Pair Test and Dot Locations from the CMS, across the different measures. Fisher’s tests were computed to compare coefficient correlations. In general, the scores of the Pair Test are more strongly correlated with those of the Word Pairs subtest than those of the Dot Locations subtest. For delayed recognition, the comparison could not be examined because this measure is not tested in the Dot Locations subtest.

### Test reliability

Exploratory factor analyses were computed separately for measures of learning, delayed recall and delayed recognition (Table 4). Across analyses, sampling adequacy measured by the Kaiser–Meyer–Olkin index was 0.82–0.88, and Bartlett’s test of sphericity was significant (*p* < 0.001), both indicating the appropriateness of interpreting factor analyses. The analyses yielded only one factor, with 52–54% of total variance explained for measures of learning, delayed recall and delayed recognition. The factor loadings for the subtests of the CMS are lower than for the subtests of the Pair Test, across the three measures. For example, 10% and 38% of variance in delayed recall scores of the Dot Locations and Word Pairs subtests can be explained by the factor, whereas higher variance in all subtests of the Pair Test (48–65%) can be explained by the factor. The factor analysis was repeated with the inclusion of FSIQ, and once again the results yielded just one factor, with only 20% of FSIQ variance explained by the factor.

**Table 4:** Factor analysis on measures of learning, delayed recall and delayed recognition

		Learning		Delayed recall		Delayed recognition	
		Factor loadings	% of Variance explained	Factor loadings	% of Variance explained	Factor loadings	% of Variance explained
Pair Test	Spoken Words	0.68	47	0.69	48	0.59	35
	Written Words	0.73	54	0.76	57	0.81	66
	Objects	0.74	55	0.81	65	0.82	67
	Designs	0.75	55	0.76	58	0.61	37
	Pseudowords	0.71	51	0.73	54	0.62	39
CMS	Dots	0.46	21	0.31	10	NA	NA
	Words	0.65	42	0.62	38	0.45	20

Additionally, the results indicated Cronbach’s *α* of 0.85, 0.86 and 0.80 for measures of learning, delayed recall and delayed recognition, respectively. Table 5 shows how Cronbach’s α would change if specific items were removed from the analysis. None of the subtests would increase the reliability of the paradigm if deleted from the analysis, suggesting that they each contribute to the reliability of the paradigm.

**Table 5:** Cronbach's α if item deleted for each subtest of the Pair Test

	Learning	Delayed recall	Delayed recognition
Spoken Words	0.82	0.84	0.80
Written Words	0.81	0.83	0.78
Objects	0.81	0.82	0.73
Designs	0.81	0.84	0.72
Pseudowords	0.82	0.84	0.78

### Test–retest reproducibility

The subgroup of 19 participants included in the test–retest analysis differed from the main sample population only in terms of sex (*p* = 0.03, independent-samples Mann–Whitney *U* test). Age at testing and FSIQ did not differ significantly (*p* = 0.6 and *p* = 0.5, respectively, two-sample *t*-test). The ICC for learning, delayed recall and delayed recognition is 0.66, 0.73 and 0.51, respectively.

### Capturing a wide range of abilities

Table 6 illustrates the percentages of participants who demonstrate floor and ceiling effects for each subtest. Floor effects are observed in 0–3.2% of participants across subtests, and ceiling effects are observed in 0–5.5%.

**Table 6:** Proportion of floor and ceiling effects for each subtest of the Pair Test (%)

	Floor effect	Ceiling effect
Spoken Words	0	0.8
Written Words	0.8	5.5
Objects	2.3	2.3
Designs	0	0
Pseudowords	3.2	0

Similar analyses were conducted for the subtests of the CMS. A high percentage of participants obtained a ceiling effect on the Dot Locations subtest, with 22% of children obtaining 100% correct performance at the first trial (Table 7A). This ceiling effect was further explored in different age groups, and results indicate that the percentage of participants obtaining at or near ceiling scores is high even in younger children (Table 7B).

**Table 7:**
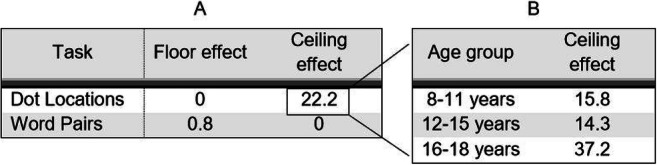
A. Percentage of floor and ceiling effects for each subtest of the CMS (%). B. Percentage of ceiling effects for Dot Locations in three age groups (%)

### Developmental changes

Table 8 illustrates partial Pearson correlation coefficients between age and measures of learning, delayed recall and recognition across the five subtests. The results show significant moderate correlations, with better scores in older children. This was observed across all subtests, apart from recognition scores for Spoken Words, which did not reach acceptable levels of statistical significance (*p* = 0.091), probably due to high performance levels across all ages. See supplementary Table 1 for the descriptive statistics of each subtest, for each measure, across the five age groups.

**Table 8:** Correlation coefficient between Pair Test and age, with FSIQ partialled out, for learning, delayed recall and delayed recognition scores

	Learning	Delayed recall	Delayed recognition
Spoken Words	0.28**	0.30**	0.16
Written Words	0.37**	0.37**	0.26**
Objects	0.45**	0.46**	0.30**
Designs	0.51**	0.52**	0.32**
Pseudowords	0.42**	0.43**	0.26**

**Correlation is significant at the 0.01 level (two-tailed)

*Correlation is significant at the 0.05 level (two-tailed)

### Variance explained by FSIQ

Regression analyses were first computed on an overall measure (scores from all processes and subtests combined) for the Pair Test and the CMS, separately, then computed for each subtest and each process separately. Significant regression was found for the Pair Test (*F*(1,128) = 15.7, *p* < 0.001), with an *R*^2^ of 0.10, and for the CMS (*F*(1,128) = 35.9, *p* < 0.001), with an *R*^2^ of 0.21. FSIQ therefore explains 10% and 21% of the memory scores on the Pair Test and the CMS, respectively. Table 9 illustrates the *R*^2^ values for each subtest of the Pair Test and the CMS, for separate processes. Scores on the CMS, and particularly on the Word Pairs subtest, can be explained by FSIQ to a larger extent than scores on the Pair Test.

**Table 9:** Variance explained by FSIQ (*R*^2^) for each subtest of the Pair Test and the CMS separately, and for different processes

		Learning	Delayed recall	Delayed recognition
Pair Test	Spoken Words	0.05	0.04	0.02
	Written Words	0.05	0.07	0.01
	Objects	0.05	0.05	0.02
	Designs	0.07	0.06	0.02
	Pseudowords	0.09	0.09	0.06
CMS	Dots	0.07	0.07	N/A
	Word Pairs	0.21	0.08	0.01

## Discussion

The aim of the present study was to establish the utility of the Pair Test as a tool to examine learning and memory in children. Although neuropsychological tests are rarely process-pure, the Pair Test does achieve a comparative degree of specificity inasmuch as it targets the core feature of hippocampal-dependent cognitive memory, which is the binding of two arbitrary items to create a new representation across the stages of encoding, learning and retrieval.

This study examined test validity, reliability and reproducibility. We also examined the utility of this novel tool as a developmentally sensitive measure of learning and memory functions, and the utility of the test across a wide age range. The specificity of the tool for measurements of learning and memory, with limited contributing factors from other cognitive processes, is also discussed. Finally, we discuss the clinical utility of this novel tool.

### Test validity

We demonstrated evidence of convergent validity of the Pair Test through significant correlations with a standardised test of memory (i.e. the CMS), and confirmed that the test measures what it intends to measure (Pawlowski et al., 2013). Paired-associate paradigms measure how well participants bind two arbitrary items in memory, and prove to be a sensitive measure of encoding, learning and retrieval after a delay. However, these correlations are moderate, probably due to other factors such as the variance explained by FSIQ and the depth of processing at encoding (see section Test specificity).

Importantly, stronger correlations were found with the Word Pairs subtest than the Dot Locations subtest of the CMS, showing that the Pair Test is more closely related to other measures of paired-associate learning than to a measure of spatial memory. Another contributing factor for this finding relates to the memory load. The memory load is greater in the paired-associate tests, wherein each item is paired with another item requiring the binding of the two items, compared to the Dot Locations test which requires the binding of the same items with a different location, thereby reducing the load. The pattern of correlations indicates adequate convergent validity with the CMS for measures that involve cued recall of paired associates. As such, the underlying process of binding items in cognitive memory is established.

In addition, no significant difference was observed between the two paradigm versions (A and B), thus allowing comparable assessment across two time points. These two analyses indicate that the pattern of validity holds both between and within tests.

### Test reliability

Test reliability was explored by examining internal consistency using Cronbach’s alpha, as well as by examining the factor structure and dimensionality of the instrument using factor analysis (Embretson, 2007; Schmitt et al., 2011). Internal consistency of the Pair Test was confirmed (α = 0.84), demonstrating high reliability, as values above 0.8 are considered acceptable for cognitive tests (Kline, 1999). Moreover, the factor analysis showed that the factor loadings for the subtests of the Pair Test (35–67%) were higher than for the subtests of the CMS (10–42%). These findings suggest that the core factor relates to mnemonic functions measured by the paired-associate learning paradigm. These functions are common across the Pair Test and the Word Pairs subtest of the CMS with relatively high factor loadings (20–67%), but not involved in the Dot Locations subtest of the CMS, which shows considerably lower factor loading (10–21%). Irrespective of differences in modality and material across tests, there is a common core factor, which we identify as paired-associate learning. Similar factor analyses stated in the CMS manual show relatively poor statistics for a one-factor model, as conducted here, compared to a multiple-factor model. This indicates that the subtests of the CMS do not converge towards a unitary general factor, in contrast to the Pair Test.

Similar factor analyses stated in the CMS manual show relatively poorer statistics for a one-factor model, as conducted here, compared to a multiple-factor model. This indicates that the subtests of the CMS do not converge towards a unitary general factor, in contrast to the Pair Test. The common factor identified across the subtests of the Pair Test allow for adequate comparisons between subtests and, in turn, comparisons between material type, input modality and levels of semantic structure.

### Test reproducibility

Test reproducibility, as defined by the consistency of performance across two time points, was examined using ICC, which indicated moderate test–retest reliability across all measures, according to the criteria suggested by Koo and Li (2016). ICC was highest for the measure of delayed recall (ICC = 0.73), which is a highly valuable measure for use in clinical practice. The CMS manual reports good test–retest reliability (*r* = 0.89). However, this measure was calculated based on Pearson correlations rather than ICC, and is therefore likely to have overestimated the reproducibility of the measure. Whereas the ICC reflects both the degree of correlation and agreement between measures (Bruton et al., 2000; Koo and Li, 2016), the Pearson correlation coefficient is only a measure of correlation and is therefore not an optimal measure of reliability (Koo and Li, 2016). Moreover, the delay between sessions was longer for the Pair Test (14 months) than for the CMS (2 months), and was therefore more susceptible to developmental changes, possibly affecting test–retest reliability over the longer time period. Change in scores of memory tests is common in children (Brooks et al., 2017), but the present study demonstrates adequate test–retest reproducibility of the Pair Test despite long time intervals.

### Capturing developing processes

Despite the large age range in the current normative cohort, the Pair Test was able to measure performance across the broad range of ability without resulting in performance limitations at the bottom and top of the scales (commonly referred to as floor and ceiling effects, respectively). The Dot Locations subtest of the CMS, on the other hand, showed a high incidence of ceiling effects (22%), even in younger children. Typically developing children and adolescents do show a large range of inter-individual variation in memory performance due to age-related changes in mnemonic strategies and emerging executive functions (Baddeley and Hitch, 1974; Baddeley, 2000; Baddeley et al., 2011; Harel et al., 2014). Uttl (2005) discussed the adverse effects of low ceilings in commonly used memory tests, such as the verbal paired-associate test from the Wechsler Memory Scale (WMS, Wechsler, 1945, 1987; Wechsler, 1997, 2008). Such a low ceiling effect leads to diminished reliability and validity (Uttl, 2005), and may underestimate cognitive impairment. The Pair Test, however, can reflect high variability in performance across individuals, permitting its use with both low- and high-functioning children and adolescents.

Moreover, age-related changes in learning, delayed recall and recognition were observed across subtests (apart from recognition of spoken words), suggesting the ability of the Pair Test to chart the development of memory functions across childhood and adolescence. The tool can be used across a wide developmental age span to measure memory development as a function of age, and chart the developmental trajectory of different memory processes. As such, the Pair Test characterises the processes underlying learning and memory during development and contributes to improved understanding of the mechanisms of these processes in the developing brain.

### Test specificity

Although intellectual status contributes to learning and memory functioning in typically developing children (Cohen, 1997), only a limited amount of the variance in the scores of the Pair Test is explained by FSIQ, as the test was designed to target newly formed representations, with reduced contribution from intellectual status.

The regression analyses show that FSIQ explains more variance in CMS scores than in Pair Tests scores. In healthy children, executive function contributes to performance on episodic memory tasks, particularly for recall rather than recognition (Rajan et al., 2014), reflecting the use of strategic processes required to encode and retrieve episodic traces (Schneider and Pressley, 1997). Tasks measuring the efficiency of stimulus-binding often require a combination of several cognitive processes, including memory and executive functions (Frischkorn and Schubert, 2018). Higher intellectual functioning is associated with the use of strategies to process information (Kron-Sperl et al., 2008). However, the Pair Test involves deep encoding of the stimulus pairs by asking the participants to make a preference judgement for each pair, thus minimising inter-individual variability in aspects of executive functions such as attention or concentration (Baumeister et al., 2007). In contrast to the Pair Test, the word pair task of the CMS does not explicitly control for the encoding procedure. Performance on that test therefore not only reflects the ability to bind and remember the pairings, but also relies on attentional control processes at encoding.

Finally, input modality can also contribute to the specificity of the Pair Test. Thus, with the exception of the Spoken Words subtest, the remainder of the Pair Test subtests rely far less on processing within the auditory modality compared to the subtests of the CMS. It is recognised that auditory input may be associated with increased cognitive load to maintain representations in temporal order (Kayser et al., 2003), and therefore increased effort (Janczyk et al., 2018). Cognitive load was also lessened in the Pair Test by the inclusion of two “easy” paired associates (i.e. semantically related). This addition makes the test more accessible to younger children, and less dependent on intellectual ability to process and maintain high cognitive load.

Through the combined influence of (i) depth of processing at encoding, (ii) input modality and (iii) cognitive load, the present findings suggest that the two processes (intellectual ability and memory) are less closely related in the Pair Test than in the CMS. The Pair Test therefore provides a more targeted measure of learning and memory than the CMS.

### A computerised test

The computerised characteristic of the Pair Test has multiple advantages associated with the optimisation of test administration. First, the use of a tablet-based application allows for a more controlled administration process with, for example, standardised presentation of stimulus pairs. Second, a portable neuropsychological tool reduces the amount of materials usually needed for standardised tests, and facilitates administration in different settings. Finally, the utilisation of the interactive platform allows for a child-friendly psychological assessment, enabling examinees to be more engaged and highly motivated.

### Clinical implications

The Pair Test may provide better-informed results by characterising the extent of memory dysfunction associated with temporal lobe pathology. First, this test could be used to characterise the status of memory in relation to different aspects of cognitive function and identify the selectivity of impairment versus a global pattern of cognitive dysfunction. Second, the balanced characteristics of the test allow one to establish the pattern of lateralisation of function and identify material-specific impairment (Hamberger et al., 2019). We outlined in section 1.3 some of the issues related to the constructs of standardised tests which prevent the investigation of memory lateralisation. To date, the use of unbalanced standardised tests has made it difficult to address the question of memory lateralisation. Even though a non-lateralised profile driven by increased plasticity is more often reported in children than in adults, with the Pair Test we are now in a better position than before to address the question of left and right temporal lobe-dependent memory deficits. Third, the assessment can relate selective deficits in learning and memory to the neural systems subserving mnemonic functions, allowing clinicians and researchers to relate cognitive symptoms to underlying brain pathology. Fourth, the assessment can be used to predict learning and memory outcome post-intervention, for example after surgical removals.

## Limitations and future directions

The representativeness of the study population needs to be considered in light of the generalisability of the norms reported in this study. Those who are willing to participate in such a research study are more likely to represent children and adolescents from higher SES backgrounds (Shaw and Hagemans, 2015). However, SES scores from the current study population fell across the whole index range (1–10). Estimates of test–retest reproducibility are preliminary due to the small number of children who consented to return for retesting. Moreover, only two subtests of the CMS were used to compare scores on the Pair Test. This was deliberate for time-efficiency of testing, and those two subtests were selected amongst all subtests as they are the closest to the Pair Test in terms of test construct.

Further work is required to establish the clinical utility of the Pair Test by validating the tool in a sample of children with known pathology of the temporal lobes. The Pair Test application has been made available in order to facilitate its use in research and clinical practice. Its incorporation into different clinical research studies will address the robustness and replicability of the tool.

## Conclusion

The Pair Test is a computerised paired-associate learning paradigm assessing learning, delayed recall and recognition for different types of information (verbal versus non-verbal materials, auditory versus visual input modalities, and semantic versus non-semantic information). The Pair Test was designed to address questions related to hemispheric specialisation and distinct memory processes. We have addressed the efficiency of the paired-associate design to assess learning, and measures of both validity and reliability in the present study confirm that the Pair Test taps into the factor of paired-associate learning more so than other constructs. Moreover, the Pair Test caters to a large developmental age range and, as such, is sensitive to detecting variations in age differences at test. This tool can therefore be useful to chart the developmental trajectory of memory across childhood and adolescence. The parallel versions of the paradigm allow systematic comparisons between performance across two time points, enabling, for example, tracking of cognitive development.

We provide age-stratified normative data for several individual scores, as well as for indices (combined scores). The examiner is therefore able to select the scores that address specific questions. We hope that the utilisation of this tool in both clinical and research settings will improve assessments of learning and memory in children, facilitate advancements in research on lateralisation of memory function in clinical populations, and deepen our understanding of neurodevelopmental trajectories of memory.

## Electronic supplementary material

ESM 1 (DOCX 102 kb)

## Data Availability

The Pair Test application, the manual for administration and scoring, the record form, the standardised tables and other relevant documents are available on protocols.io (dx.doi.org/10.17504/protocols.io.bjvckn2w).
