# Supplementary material for: The Pair Test: A computerised measure of learning and memory
Source: Behav Res Methods. 2020 Sep 9;53(2):928–42. doi: 10.3758/s13428-020-01470-9 (PMC8062426; doi:10.3758/s13428-020-01470-9)
Supplement: Supplementary file 1 — (DOCX 102 kb) [file 13428_2020_1470_MOESM1_ESM.docx]

**Supplementary material**

**Table S.1**Descriptive statistics (mean % correct recall (SD)) for each measure of each subtest, across each age group

|  |  | 8-9y | 10-11y | 12-13y | 14-15y | 16-18y |
| --- | --- | --- | --- | --- | --- | --- |
| Learning | Spoken words | 54(19) | 55(23) | 58(19) | 56(22) | 70(21) |
|  | Written words | 50(23) | 51(25) | 68(25) | 68(25) | 76(20) |
|  | Objects | 56(21) | 53(23) | 63(24) | 59(25) | 80(13) |
|  | Designs | 27(15) | 33(17) | 43(16) | 51(22) | 59(17) |
|  | Pseudowords | 28(21) | 32(17) | 35(18) | 42(24) | 52(21) |
| Delayed recall | Spoken words | 65(24) | 66(27) | 71(24) | 70(29) | 84(21) |
|  | Written words | 58(32) | 62(30) | 80(25) | 80(25) | 87(20) |
|  | Objects | 64(25) | 61(29) | 74(25) | 71(28) | 91(11) |
|  | Designs | 43(19) | 42(22) | 49(23) | 59(23) | 73(21) |
|  | Pseudowords | 32(23) | 35(24) | 41(25) | 51(31) | 63(26) |
| Delayed recognition | Spoken words | 89(17) | 93(11) | 93(10) | 95(11) | 96(10) |
|  | Written words | 86(15) | 88(19) | 96(8) | 96(12) | 97(12) |
|  | Objects | 91(12) | 91(13) | 94(10) | 94(12) | 99(6) |
|  | Designs | 82(17) | 78(17) | 90(12) | 93(11) | 93(14) |
|  | Pseudowords | 83(21) | 83(23) | 88(15) | 85(22) | 94(9) |

**
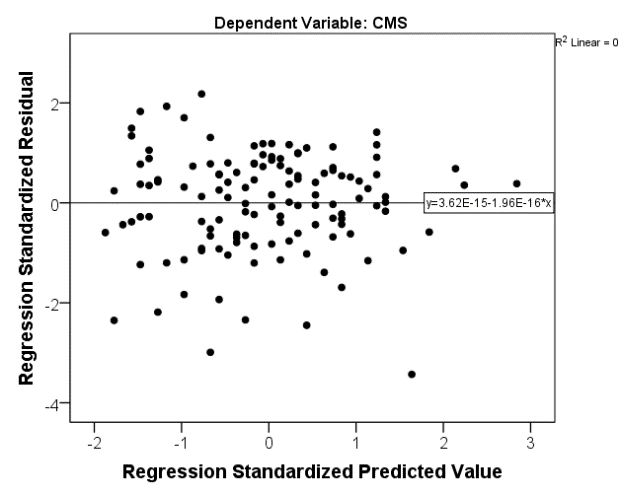

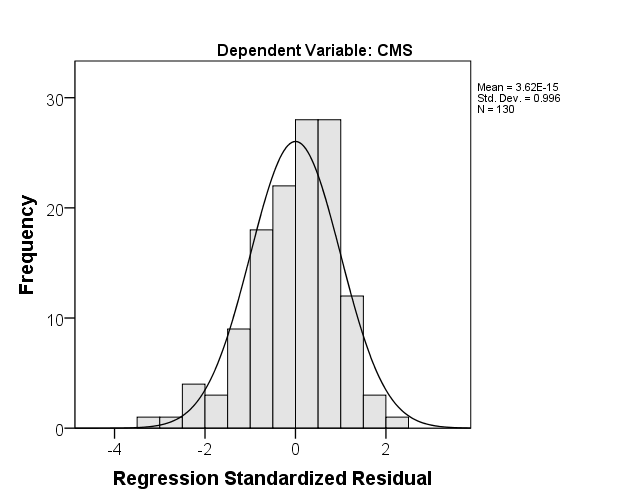

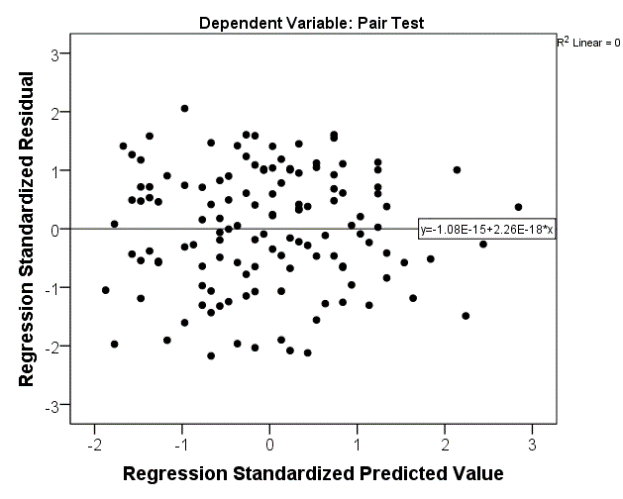

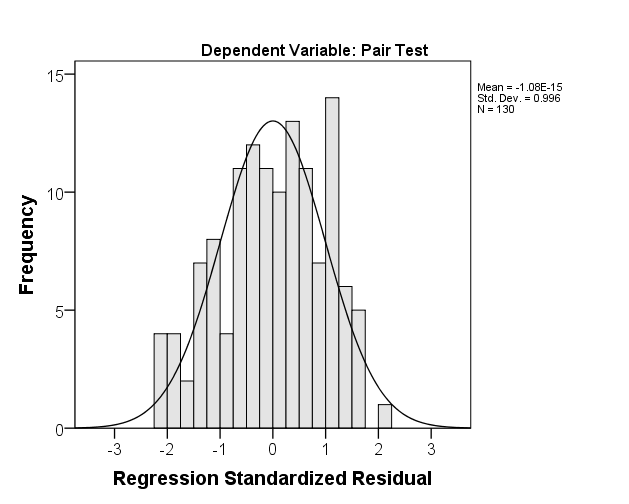
**

**Figure S.1** Normality of residuals and homoscedasticity, for the Pair Test and the CMS.
